# RETRACTION: Treatment of Wound Infections Linked to Neurosurgical Implants

**DOI:** 10.1111/iwj.70518

**Published:** 2025-04-18

## Abstract

**RETRACTION:** WangY. , WangY. , S. WangS., Hou, YuD. , ZhangC. , ZhangL. , and LinN. , “Treatment of Wound Infections Linked to Neurosurgical Implants,” International Wound Journal 21, no. 4 (2024): e14528, 10.1111/iwj.14528.38098284 PMC10961032

The above article, published online on 14 December 2023, in Wiley Online Library (http://onlinelibrary.wiley.com/), has been retracted by agreement between the journal Editor in Chief, Professor Keith Harding; and John Wiley & Sons Ltd. Following an investigation by the publisher, all parties have concluded that this article was accepted solely on the basis of a compromised peer review process. In addition, the investigation found significant textual overlap between this article and another article by different authors [1]. The editors have therefore decided to retract the article. The authors did not respond to our notice regarding the retraction.

Reference

[1] ConenA. , RaabeA. , SchallerK. , FuxC. A. , VajkoczyP. , and TrampuzA. , “Management of Neurosurgical Implant‐Associated Infections,” World Neurosurg 150, no. 1718 (2020): w20208, 10.4414/smw.2020.20208.32329803

**RETRACTION:** Y. Wang , Y. Wang , S. Wang, S. Hou, D. Yu , C. Zhang , L. Zhang , and N. Lin , “Treatment of Wound Infections Linked to Neurosurgical Implants,” International Wound Journal 21, no. 4 (2024): e14528, 10.1111/iwj.14528.38098284 PMC10961032

The above article, published online on 14 December 2023, in Wiley Online Library (http://onlinelibrary.wiley.com/), has been retracted by agreement between the journal Editor in Chief, Professor Keith Harding; and John Wiley & Sons Ltd. Following an investigation by the publisher, all parties have concluded that this article was accepted solely on the basis of a compromised peer review process. In addition, the investigation found significant textual overlap between this article and another article by different authors [1]. The editors have therefore decided to retract the article. The authors did not respond to our notice regarding the retraction.

**Reference**

[1] A. Conen , A. Raabe , K. Schaller , C. A. Fux , P. Vajkoczy , and A. Trampuz , “Management of Neurosurgical Implant‐Associated Infections,” World Neurosurg 150, no. 1718 (2020): w20208, 10.4414/smw.2020.20208.32329803

